# Enhance of tomato production and induction of changes on the organic profile mediated by *Rhizobium* biofortification

**DOI:** 10.3389/fmicb.2023.1235930

**Published:** 2023-08-02

**Authors:** Adriana Gen-Jiménez, José David Flores-Félix, Clara Ivette Rincón-Molina, Luis Alberto Manzano-Gomez, Marco Antonio Rogel, Víctor Manuel Ruíz-Valdiviezo, Francisco Alexander Rincón-Molina, Reiner Rincón-Rosales

**Affiliations:** ^1^Laboratorio de Ecología Genómica, Tecnológico Nacional de México, Instituto Tecnológico de Tuxtla Gutiérrez, Tuxtla Gutiérrez, Chiapas, Mexico; ^2^Departamento de Microbiología y Genética, Universidad de Salamanca, Salamanca, Spain; ^3^Departamento de Investigación y Desarrollo, 3R Biotec SA de CV, Tuxtla Gutiérrez, Chiapas, Mexico; ^4^Centro de Ciencias Genómicas, Universidad Nacional Autónoma de México, Cuernavaca, Morelos, Mexico

**Keywords:** microbiome, plant probiotic, quality, rhizobium species, tomato

## Abstract

**Introduction:**

The extensive use of chemical fertilizers has served as a response to the increasing need for crop production in recent decades. While it addresses the demand for food, it has resulted in a decline in crop productivity and a heightened negative environmental impact. In contrast, plant probiotic bacteria (PPB) offer a promising alternative to mitigate the negative consequences of chemical fertilizers. PPB can enhance nutrient availability, promote plant growth, and improve nutrient uptake efficiency, thereby reducing the reliance on chemical fertilizers.

**Methods:**

This study aimed to evaluate the impact of native *Rhizobium* strains, specifically *Rhizobium calliandrae* LBP2-1, *Rhizobium mayense* NSJP1-1, and *Rhizobium jaguaris* SJP1- 2, on the growth, quality, and rhizobacterial community of tomato crops. Various mechanisms promoting plant growth were investigated, including phosphate solubilization, siderophore production, indole acetic acid synthesis, and cellulose and cellulase production. Additionally, the study involved the assessment of biofilm formation and root colonization by GFP-tagged strains, conducted a microcosm experiment, and analyzed the microbial community using metagenomics of rhizospheric soil.

**Results:**

The results showed that the rhizobial strains LBP2-1, NSJP1-1 and SJP1-2 had the ability to solubilize dicalcium phosphate, produce siderophores, synthesize indole acetic acid, cellulose production, biofilm production, and root colonization. Inoculation of tomato plants with native *Rhizobium* strains influenced growth, fruit quality, and plant microbiome composition. Metagenomic analysis showed increased Proteobacteria abundance and altered alpha diversity indices, indicating changes in rhizospheric bacterial community.

**Discussion:**

Our findings demonstrate the potential that native *Rhizobium* strains have to be used as a plant probiotic in agricultural crops for the generation of safe food and high nutritional value.

## 1. Introduction

The increasing global population has resulted in an expansion of agricultural activities to meet the growing demand for food. However, this expansion poses a threat to food security due to ecosystem degradation, climate change, soil erosion, and biodiversity loss. Additionally, the excessive use of chemical fertilizers and pesticides on a large scale to increase crop production raises concerns for environmental protection (Verma et al., 2018). Therefore, the use of biofertilizers has become increasingly important in recent decades due to their high efficiency in promoting plant growth, enhancing soil fertility, and reducing environmental pollution (Mitter et al., 2021; Kumar et al., 2022). In Mexico, the utilization of biofertilizers has been on the rise, driven by the demand for sustainable and environmentally friendly agricultural practices. *Rhizobium*, *Azotobacter*, and *Azospirillum* are among the commonly employed nitrogen-fixing microorganisms (Santillano-Cázares et al., 2022). Biofertilizers hold promise in agriculture by enhancing nutrient availability, improving soil fertility, promoting sustainability, and increasing plant growth and yield. However, challenges such as specificity, quality control, adoption, limited nutrient range, and regulatory considerations exist. Further research is crucial to optimize the efficacy of biofertilizers and address these limitations, thereby maximizing their potential in sustainable agricultural practices. Biofertilizers are formulated inoculants consisting of live or dead bacteria, algae, fungi, or combinations thereof, that enhance plant growth and nutrition by increasing the availability of nutrients in soil (Kumar et al., 2022). Plant probiotic bacteria (PPB) are commonly used for formulating biofertilizers. PPB are beneficial microorganisms that colonize the root system of plants and have the ability to promote plant growth and health. These bacteria are capable of interacting beneficially with the host plant through mechanisms such as the production of phytohormones, atmospheric nitrogen fixation, phosphate solubilization, and the production of enzymes and secondary metabolites with antibacterial and antifungal properties (Mahanty et al., 2017). The application of plant probiotic bacteria (PPB) can have positive effects on the yield and quality of vegetable crops. Overall, the use of PPB can improve crop productivity, reduce the need for synthetic fertilizers and pesticides, and promote sustainable agricultural practices. Moreover, these microorganisms are recognized as a useful means to produce highly functional foods that enhance human health through the increased presence of bioactive compounds and the replacement of chemical fertilizers with biofertilizers (Glick, 2012; Flores-Félix et al., 2015; Olanrewaju et al., 2017; Suvega and Arunkumar, 2019; Anzalone et al., 2021). However, only microorganisms safe for humans and plants should be used as biofertilizers, due to plants or their fruits may be intended for raw consumption, in order to avoid sanitary problems derived from the presence of pathogenic bacteria in the final products (Anzalone et al., 2021). Some of the most commonly used as biofortifiers due to their safety and innocuousness belong to the genera *Bacillus*, *Pseudomonas*, and *Rhizobium*. The genus *Rhizobium* is a phylogenetically diverse group of symbiotic nitrogen-fixing rhizospheric bacteria that are capable of surviving even without associating with plant hosts. These bacteria have been widely studied because, in addition to being harmless to health, they have functional qualities as plant growth-promoting bacteria (PGPB) and also as plant probiotics (Tiwari et al., 2019). *Rhizobium* are considered excellent plant probiotics bacteria due to their ability to establish a symbiotic relation (Gómez-Godínez et al., 2023). In addition, many *Rhizobium* species have been shown to colonize the rhizosphere of non-leguminous plants and promote their growth and disease resistance, making them an attractive option as biofertilizers for a wide range of crops (García-Fraile et al., 2012; Flores-Félix et al., 2019; Kang et al., 2019; Kumar et al., 2020). For instance, studies have demonstrated the beneficial impact of specific strains such as *Rhizobium leguminosarum* PETP01 and TVP08 on pepper (Silva et al., 2014), *R. laguerreae* PEPV40 on spinach (Jiménez-Gómez et al., 2018), and *R. leguminosarum* PEPV16 on lettuce and carrot (Flores-Félix et al., 2021). Plants inoculated with these strains produced significantly higher numbers of fruits with greater fresh weight, and the fruits reached the maturation stage earlier. On spinach crops increased vegetative parameters, plus the content of chlorophyll and nitrogen in the leaves (Jiménez-Gómez et al., 2018). The inoculation of *Rhizobium* has been shown to promote plant growth and increase the dry matter of shoots and roots in lettuce and carrot crops (Flores-Félix et al., 2013). In addition, it has been found to increase the uptake of nitrogen and phosphorus in the edible parts of both plant species. Furthermore, in corn, the inoculation of *Rhizobium* resulted in increased plant traits such as the number of leaves per plant, number of seeds per row, number of seeds per ear, plant height, and forage yield (Naserzadeh et al., 2019). Rincón-Rosales et al. (2013) reported the effect of the inoculation of native nitrogen-fixing strains *Rhizobium calliandrae* LBP2-1, *Rhizobium mayense* NSJP1-1 and *Rhizobium jaguaris* SJP1-2 on the growth of common bean plants.

Tomato (*Solanum lycopersicum*) is a highly cultivated vegetable and a significant agricultural industry. It is versatile in consumption as a fresh or processed vegetable, and it is an excellent dietary source of antioxidant compounds, vitamins A, C, E, and carotenoids, with beneficial effects on human health, making it popular worldwide. Therefore, it is a promising candidate for biofertilization with plant probiotics (Randome et al., 2017).

Introducing new strains into an ecological niche requires careful consideration of their potential impact on preexisting communities. Recent studies have highlighted the variability in the effects of plant growth-promoting bacteria (PGPB) inoculation on soil microbial diversity (Ren et al., 2019). The efficacy and prevalence of PGPB depend on their versatility, adaptability to environmental changes, and ability to colonize and compete with other members of the microbial community. When a new strain is introduced into the rhizosphere of plants, its impact on soil microorganisms can vary, as it may enhance certain groups, inhibit others, or have no effect on population structure (Tang et al., 2023). Metagenomics can provide valuable information on the phylogeny, structure, and diversity of the biological community in a natural ecosystem (Trabelsi et al., 2011; Gaiero et al., 2013; Kong et al., 2019).

In this study, we aimed to assess the potential of native *Rhizobium* strains as plant probiotic bacteria (PPB) for enhancing the growth and fruit quality of tomato plants, as well as their impact on the rhizobacterial community. To achieve this, we conducted *in vitro* and *in vivo* tests on each strain and performed metagenomic analysis of the rhizospheric soil.

## 2. Materials and methods

### 2.1. Bacterial strains

The native nitrogen-fixing strains *Rhizobium calliandrae* LBP2-1^T^ (JX855162), *Rhizobium mayense* NSJP1-1^T^ (JX855172), and *Rhizobium jaguaris* SJP1-2^T^ (JX855169) were isolated from the shrubby legume *Calliandra grandiflora* (Rincón-Rosales et al., 2013). These novel *Rhizobium* species are known for their ability as plant growth-promoting bacteria (PGPB) and possess non-toxic and non-pathogenic characteristics. The strains are maintained in the culture collection of the Genomic Ecology Laboratory at Tecnológico de Tuxtla Gutierrez, Chiapas, Mexico.

### 2.2. *In vitro* assessment of PGP mechanisms of native *Rhizobium*

#### 2.2.1. Solubilization of phosphate

Phosphate solubilization by native *Rhizobium* bacteria was assessed using Pikovskaya’s medium (PVK). The PVK medium composition per liter consisted of glucose (10 g), Ca_3_(PO_4_)_2_ (5 g), (NH_4_)_2_SO_4_ (0.5 g), NaCl (0.2 g), MgSO_4_.7H_2_O (0.1 g), KCl (0.2 g), yeast extract (0.5 g), MnSO_4_.H_2_O (0.002 g), and FeSO_4_.7H_2_O (0.002 g). The medium was supplemented with 0.2% dicalcium phosphate (Ca_2_PO_3_) or tricalcium phosphate (Ca_3_(PO_4_)_2_) as the phosphate source (Pikovskaya and Pikovskaya, 1948). After inoculation, plates were incubated at 28°C for 15 days, and halos surrounding the colonies indicating phosphate solubilization were observed. The phosphate solubilization index (PSI) was calculated by measuring the ratio of halo size to colony size. Soluble phosphorus concentration was determined using the ammonium vanadate-molybdate method at 420 nm with a UV/visible spectrophotometer. The methodology recommended by O’Halloran and Cade-Menun (2006) was followed. Bacterial isolates were cultured in 125 mL conical flasks containing 50 mL of liquid Research Institute’s phosphate growth medium (NBRIP). The NBRIP medium composition per liter included glucose (10 g), Ca_3_(PO_4_)_2_ (5 g), MgCl_2_.6H_2_O (5 g), MgSO_4_.7H_2_O (0.25 g), KCl (0.2 g), and (NH_4_)_2_SO_4_ (0.1 g). The pH of NBRIP was adjusted to 7.0. Flasks were inoculated with a 1 mL aliquot of 1 × 10^6^ cell suspensions and incubated at 30°C, 125 rpm, with monitoring every 24 h for 6 days. Non-inoculated liquid NBRIP served as the negative control. The experiment was performed in triplicate.

#### 2.2.2. Siderophore production

Qualitative and quantitative determination of siderophore production was done by CAS assay (Schwyn and Neilands, 1987). Bacterial isolates were grown in CAS-agar medium [chromeazurol-S (CAS), iron (III) and hexadecyl trimethyl ammonium bromide (HDTMA)]. Colonies exhibiting an orange halo after 3 days incubation (28°C ± 2°C) were considered positive for siderophore production and the diameter of the orange halo was measured. The result obtained was expressed as Siderophore Induced Droplet Formation (SID; Alexander and Zuberer, 1991). For siderophore quantification, *Rhizobium* strains in the logarithmic growth phase were cultured in 25 mL of King’s B broth in 125 mL flasks and incubated at 28°C for 3 days. Bacterial cells were separated by centrifugation at 3,000 rpm. A 0.5 mL aliquot of supernatant was mixed with 0.5 mL of CAS solution and 10 μL of sulfosalicylic acid. After incubation for 20 min, absorbance was measured at 630 nm using a Beckman Coulter® DU730 spectrophotometer. Uninoculated King’s B broth served as a blank. The percentage of siderophore production was calculated using the formula: (Ar − As)/Ar × 100, where Ar represents the absorbance of the reference solution and As represents the absorbance of the samples (Priyanka Agrawal et al., 2017).

#### 2.2.3. Indole acetic acid production

The quantification of IAA production in each bacterial strain was performed using the colorimetric method with Salkowski reagent. The reagent consisted of 50 mL of 35% perchloric acid (HClO_4_) and 1 mL of 0.5 M iron trichloride (FeCl_3_), following the procedure proposed by Bric et al. (1991). Bacterial isolates were cultured in 250 mL Erlenmeyer flasks containing 50 mL of YMB medium, supplemented with 2 g/L (w/v) L-tryptophan. YMB medium contained per liter: 5 g yeast extract, 10 g mannitol, 0.2 g KH_2_PO_4_, 0.1 g K_2_HPO_4_, 0.2 g MgCl_2_, 0.2 g MgSO_4_·7H_2_O, 0.02 g CaCl_2_, and 0.01 g phenol red, with a pH adjusted to 7.0. The cultures were incubated at 28°C ± 2°C for 7 days at 150 rpm in an orbital shaking incubator. After centrifugation at 10,000 rpm for 10 min at 4°C, the supernatant was mixed with Salkowski reagent at a ratio of 1:2. The mixture was incubated for 30 min in the dark at 28 ± 2°C, and the absorbance was measured at 530 nm (O’Hara et al., 1989). The concentration of IAA produced was determined using a standard IAA curve. All IAA determination experiments were conducted in triplicate.

#### 2.2.4. Cellulose production

Cellulose production by native *Rhizobium* strains was assessed using Yeast Extract Mannitol (YEM) medium (10 g mannitol, 0.5 g K_2_HPO_4_, 0.2 g MgSO_4_, 0.1 g NaCl, 3.0 g CaCO_3_, and 3.0 g yeast extract per 10 g, pH 6.8), supplemented with 0.25% Congo Red. Congo Red, a dye that binds to β 1–4 bonds found in cellulose-like polysaccharides, enabled the visualization of red-stained bacterial colonies. The inoculated plates were incubated at 28°C for 5 days and observed using a Zeiss® Stemi 2000-C Stereo Microscope to detect stained colonies (Robledo et al., 2012).

#### 2.2.5. Cellulase activity

Cellulase activity was determined on plates containing YEM medium supplemented with carboxymethylcellulose 1% (Hankin and Anagnostakis, 1977). The inoculated plates were incubated for 7 days at 28°C. Colonies were then removed with distilled water and plates were stained with a 0.1% Congo Red solution for 30 min. Washes were carried out with NaCl 1 M until the lysis halos showing the production of celluloses were visualized (Flores-Félix, 2018).

### 2.3. Evaluation of biofilms formation ability

#### 2.3.1. Quantitative determination

The ability of native strains *R. calliandrae* LBP2-1, *R. mayense* NSJP1-1, and *R. jaguaris* SJP1-2 to form biofilms on abiotic surfaces was assessed using the microtiter plate assay with crystal violet post-staining (Robledo et al., 2012). The strains were cultured in minimal medium until reaching a value of 0.2 OD_600_ (1 × 10^8^ UFC/mL). Subsequent biofilm formation measurements were performed using decimal dilutions. Biofilm data were analyzed using one-way ANOVA, followed by Fisher’s *post hoc* test (*p* < 0.05), with StatView 5.0 software (Abacus Corporation, Berkeley, CA, United States). Statistical significance was determined using one-way ANOVA, and mean values were compared using Fisher’s Protected LSD test (Least Significant Differences; *p* < 0.05).

#### 2.3.2. Qualitative determination

Biofilm formation by native *Rhizobium* strains was evaluated in Falcon tubes containing 25 mL of liquid YEM medium. A sterile slide was inserted, and the tubes were inoculated with 48-h-old strains grown on YEM plates. Incubation was carried out for 5 days at 28°C, with a 45° inclination and shaking at 150 rpm. The slides were stained with acridine orange and examined using a Nikon Eclipse i80 fluorescence microscope following the recommendations of Flores-Félix et al. (2015).

### 2.4. Root colonization capacity assay

One lot of 50 tomato (*Solanum lycopersicum*) var. Rome seeds was surface-sterilized by treating with 70% ethanol for 30 s, followed by soaking in a 5% aqueous solution of sodium hypochlorite for 5 min. The seeds were then rinsed five times with sterile water and germinated on water-agar plates. After germination, 25 healthy and vigorous seedlings were selected and kept in darkness for 3 days, and then transferred to 1% agar square plates (12 × 12 cm) with five seedlings per plate. The plants were inoculated with GFP-tagged *Rhizobium* strains to assess their colonization effect (Cheng and Walker, 1998). For preparing the bacterial suspensions, the strains were grown on YMA plates at 28°C for 5 days, and then flooded with sterile water to obtain the suspensions, which were transferred to sterile flasks. The suspensions were adjusted to an optical density (OD) of 0.2 at 600 nm, corresponding to a final concentration of 1×10^8^ CFU/mL. Using a micropipette, each seedling was inoculated with 250 μL of this suspension at the junction between the roots and cotyledons. The plants were kept in a growth chamber and observed at 5, 7, 12, and 15 days post-inoculation (dpi). Before microscopic observation, the roots were gently washed three times with sterile distilled water. Fluorescence microscopy was performed using a Nikon Eclipse 80i, and green fluorescent protein excitation was achieved using a mercury lamp (Jiménez-Gómez et al., 2018).

### 2.5. *In vivo* assays in microcosm experiments

In this experiment, the effect of inoculation with native *Rhizobium* strains on tomato plant growth and fruit quality were evaluated. Five treatments were applied: T_1_: *Rhizobium calliandrae* LBP2-1, T_2_: *Rhizobium mayense* NSJP1-1, T_3_: *Rhizobium jaguaris* SJP1-2, T_4_: Chemical fertilizer (Nitrabor®), and T_5_: Negative control (plants without inoculum or fertilizer). Forty non-sterilized seeds were germinated for each treatment and irrigated with water every 48 h and Farhaeus nutrient solution (Fahraeus, 1957) every 8 days. Each seed was placed in a polyethylene agricultural bag filled with a substrate of peat moss and agrolite (2:1) and inoculated with 1 mL of a 5-day-old *Rhizobium* strain suspension (1 × 10^8^ CFU/mL) grown in YMA at 28°C. For the fertilizer treatment, 5.0 mL of a 1.5% Nitrabor solution was applied per pot. The pots were covered and germinated under greenhouse conditions using a randomized design. After 4 weeks, 20 plants per treatment were analyzed for shoot height, root length, fresh and dry root weight, and chlorophyll content. The remaining 20 plants per treatment were transplanted into pots and immersed in 100 mL of a bacterial suspension (1 × 10^8^ CFU/mL) for 4 h. Each plant was transplanted into a pot with 2 kg of agricultural soil and irrigated accordingly. The plants were grown in a greenhouse under natural light during spring until fruit was obtained. Fruit weight and quantity per plant were recorded. The potassium content was analyzed using Inductively Coupled Plasma–Optical Emission Spectroscopy (ICP-OES) (González-Terreros et al., 2018). For lycopene and carotenoid analysis, the pigments were extracted using acetone and measured at 450 nm using a spectrophotometric method (Ceballos-Aguirre et al., 2012). A calibration curve was constructed using standard solutions to determine concentrations in the samples. Results were expressed as micrograms per gram of sample. The experiments were performed in triplicate and presented as mean ± standard deviation. Data were analyzed using one-way analysis of variance with Fisher’s Protected Least Significant Differences (LSD) test (*p* < 0.05) using the Statgraphics Centurion V. 15.2 program.

### 2.6. Study of microbial communities

The structure and composition of bacterial communities associated with tomato plants inoculated with rhizobial bacteria were investigated through metagenomic analysis (Sessitsch et al., 2012). Rhizospheric soil DNA was extracted using the DNeasy PowerSoil Pro Kit (QIAGEN), and its concentration was quantified using a NanoDrop 2000 spectrophotometer. The V1–V3 hypervariable regions of the 16S rRNA gene were amplified for each metagenomic DNA sample through PCR. The PCR products were subsequently sequenced using the Illumina MiSeq 2000 platform by Macrogen®. Sequence alignments were performed against the Greengenes core set using PyNAST, and a filtering threshold of 75% was applied (Caporaso et al., 2010). Taxonomic assignation was conducted using the naïve Bayesian rRNA classifier from the Ribosomal Data Project at an 80% confidence threshold (Wang et al., 2007). Operational taxonomic units (OTUs) were clustered at a 97% shared similarity, representing the species level classification. In cases where complete lineage information was available in the database, the genus name was maintained as the most specific rank. Otherwise, the deepest rank achieved among domain, phylum, class, order, or family was reported. The raw sequence data obtained in this study were deposited in the Sequence Read Archive (SRA) database at NCBI under the accession number PRJNA940440. Bioinformatic analysis was performed using the Microbiome Analyst package (Chong et al., 2020). To evaluate the diversity and abundance of the bacterial community, the relative abundances of taxa were first normalized using the total sum of squares scaling (CSS). OTUs coming from amplification of chloroplast DNA were discarded from the downstream analyses. The Chao1, ACE, Shannon, and Simpson indices were employed for evaluating diversity (Rincón-Molina et al., 2022). Beta-diversity was assessed using PCoA based on the Bray-Curtis Index, and statistical significance was determined using the *T*-test and Permutational Multivariate Analysis of Variance (PER-MANOVA). Additionally, the Pearson Correlation Index was utilized to examine the relationship between taxa, treatment, and variations in their abundances.

## 3. Results

### 3.1. Plant growth promotion ability of native *Rhizobium* strains

The strains *R. calliandrae* LBP2-1, *R. mayense* NSJP1-1 and *R. jaguaris* SJP1-2 have the ability to solubilize dicalcium phosphate; however, they are not capable of solubilizing tricalcium phosphate (Supplementary Figure S1). The *R. mayense* NSJP1-1 strain had the highest index of solubilization of phosphate (PSI = 1.15) compared to the other rhizobial strains (Table 1). In the quantitative tests utilizing the ammonium vanadate-molybdate method, it was determined that the native *Rhizobium* strains possess the biochemical capacity to solubilize phosphate, including both dicalcium phosphate (Ca_2_PO_3_) and tricalcium phosphate (Ca_3_(PO_4_)_2_). Among the strains tested, *Rhizobium jaguaris* SJP1-2 exhibited the highest biochemical capacity for solubilizing both types of inorganic phosphates (Table 1).

**Table 1:** Phosphate solubilization, siderophore production, and indole acetic acid (IAA) production of native *Rhizobium* strains.

Strain	P-solubilization			Siderophore production		IAA production (mg/L)
	PSI^¥^	Ca_2_PO_3_ (mg/L)	Ca_3_(PO_4_)_2_ (mg/L)	SID^≠^	% siderophore units	
*Rhizobium calliandrae* LBP2-1	1.12 ± (0.21)^*^	48.5 ± (1.17)	22.1 ± (1.1)	1.05 ± (0.05)	54.7 ± (1.76)	21.0 ± (0.41)
*Rhizobium jaguaris* SJP1-2	1.10 ± (0.32)	62.2 ± (2.14)	34.7 ± (1.18)	1.44 ± (0.04)	22.4 ± (1.92)	35.0 ± (0.82)
*Rhizobium mayense* NSJP1-1	1.15 ± (0.26)	54.4 ± (2.09)	25.8 ± (1.09)	1.28 ± (0.02)	12.4 ± (1.12)	42.0 ± (0.62)

* Mean values of three replicates. The values in parenthesis are standard deviations.

^¥^PSI, Phosphate Solubilization Index. ^≠^SID, Siderophore Induced Droplet Formation.

With respect to siderophores production, was observed that the three strains produced halos that indicate the presence of this PGP mechanism (Supplementary Figure S1), and the *R. jaguaris* SJP1-2 and *R. mayense* NSJP1-1 strains have the highest production index (SID = 1.44, and SID = 1.28, respectively). Similarly, in the quantitative assay for siderophore production, it was observed that all three *Rhizobium* strains exhibited the ability to produce this crucial metabolite. Among the *Rhizobium* species tested, the *R. calliandrae* LBP2-1 strain demonstrated the highest percentage of siderophore production (54.7%; Table 1). Likewise, strains *R. calliandrae* LBP2-1, *R*. *jaguaris* SJP1-2, and *R. mayense* NSJP1-1 had the ability to synthesize auxins, such as indole-3-acetic acid (IAA) in a range of 21 to 42 mg/L (Table 1). The strain NSJP1-1 stood out for its high production of IAA (42 mg/L).

Congo red is a compound that has the ability to bind to the β-1,4 bonds found in cellulose, which are responsible for linking glucose molecules. Therefore, the reddish coloration of the colonies indicates the presence and relative abundance of cellulose. Positive results are indicated by shades ranging from pink to intense red. In this assay, *R. jaguaris* SJP1-2 displayed a more pronounced reddish hue, whereas the colonies of *R. calliandrae* LBP2-1 and *R. mayense* NSJP1-1 appeared practically white, indicating a lack of cellulose production by these two bacteria (Supplementary Figure S1).

In addition, *R. calliandrae* LBP2-1 follow by *R. mayense* NSJP1-1 were the strains which presented greater cellulase enzyme activity. Although these strains do not produce cellulose, but had lytic activity, which could help the strain in the colonization of roots (Supplementary Figure S1). On the other hand, even though *R. jaguaris* SJP1-2 had a weak lytic activity, it is able to produces cellulose, which gives it the ability to form exopolysaccharides.

### 3.2. Colonization capacity by native *Rhizobium*

#### 3.2.1. Biofilms formation

**Figure 1:**
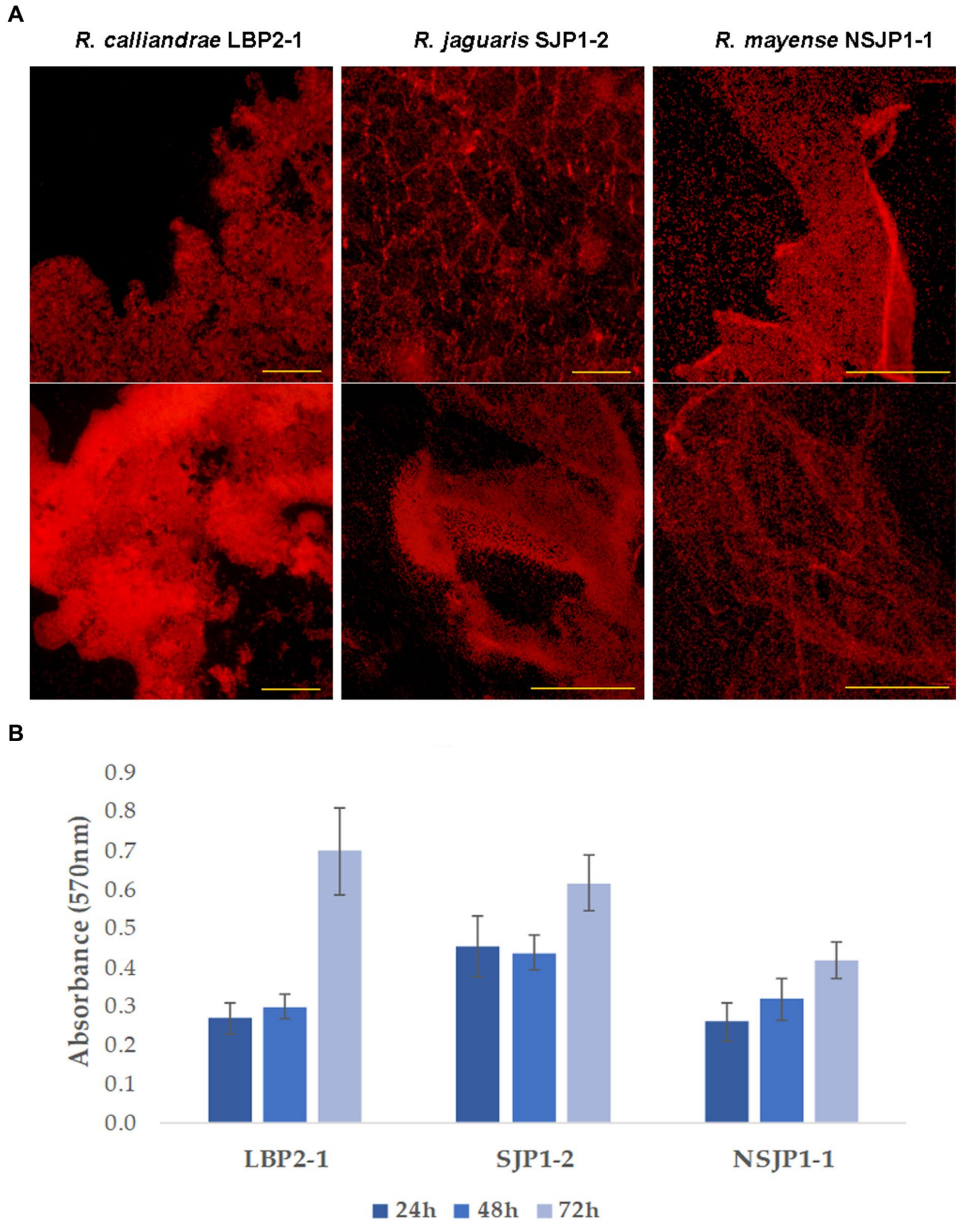
Biofilms production by native *Rhizobium*. **(A)** Fluorescence optical micrographs of biofilms production. **(B)** Production of biofilms expressed relatively in absorbance units at 570 nm.

The colonization capacity was evaluated by bacterial biofilm formation qualitatively and quantitatively. On qualitative assay, two different colonization patterns were observed, the first consisted of a more intense colonization, with a great three-dimensional development and the formation of numerous imbrications developed by *R. calliandrae* LBP2-1, in the case of *R. jaguaris* SJP1-2 large surfaces and less dense accumulations than the previous one are observed; the second colonization pattern is observed with *R. mayense* NSJP1-1, which consists of a diffuse pattern with a veil-like conformation with multiple folds (Figure 1A). In quantitative results, a similar behavior is observed in the dynamic production of biofilms in the strains *R. calliandrae* LBP2-1 and *R. jaguaris* SJP1-2 where during the first 48 h the production remains constant and after 72 h it doubles. While, in the case of *R. mayense* NSJP1-1 the production has a linear behavior, and the yield of the production is lower compared to the other strains (Figure 1B). These differences in biofilm production dynamics are related to the production of exopolysaccharides and their characteristic.

#### 3.2.2. Colonization of tomato roots

**Figure 2:**
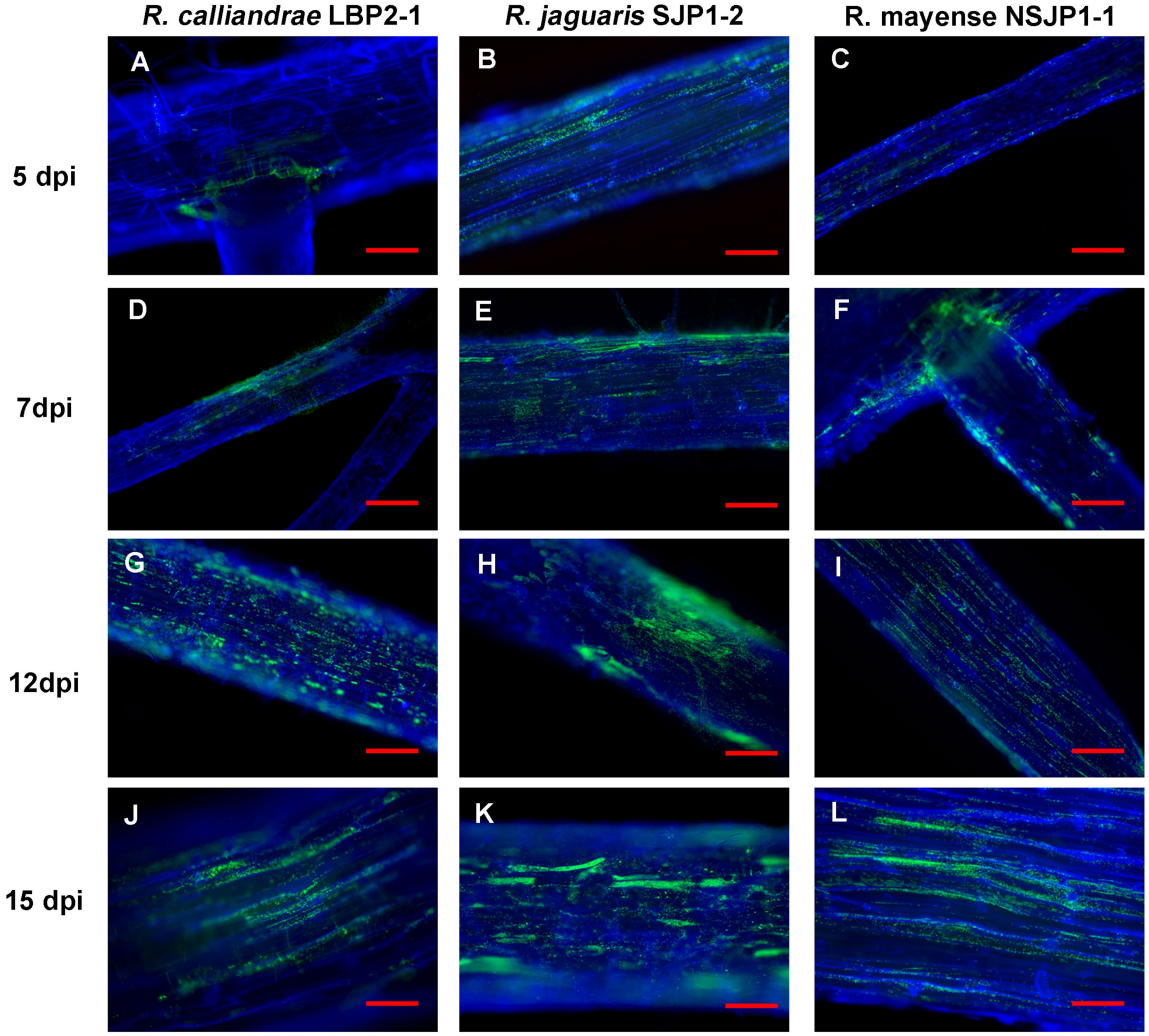
Fluorescence microscopy of tomato roots inoculated with *R. calliandrae* LBP2-1 **(A,D,G,J)**, *R. jaguaris* SJP1-2 **(B,E,H,K)** and *R. mayense* NSJP1-1 **(C,F,I,L)** at different time after inoculation.

Through the use of fluorescence microscopy, we were able to observe that native *Rhizobium* strains, tagged with GFP, exhibited the ability to adhere to the root surfaces of inoculated tomato seedlings. Additionally, this adhesion was observed to increase over time (Figure 2).

*Rhizobium calliandrae* LBP2-1 during the first 5 days post inoculation (dpi) accumulations of bacteria appeared in the basal areas of the secondary roots, although the colonization was irregular and did not cover large spaces. After 7 days, the colonization became more extensive and with a more defined pattern, at 12 and 15 days a geometric distribution was observed as shown Figures 2A,D,G,J.

*Rhizobium jaguaris* SJP1-2 showed scattered but higher colonization in the first 5 days compared to the other two strains. Colonization was found on root hairs. In later days, a colonization was observed with accumulations in certain areas along the root and following the geometric pattern of the epidermal cells of the root (Figures 2B,E,H,K).

*Rhizobium mayense* NSJP1-1 had a little intense and diffuse colonization pattern during the first days, this colonization became more localized in the following days, the bacteria were concentrated at the apical end of the roots. At 15 days, colonization was a little more uniform (Figures 2C,F,I,L).

### 3.3. Microcosm experiments

**Figure 3:**
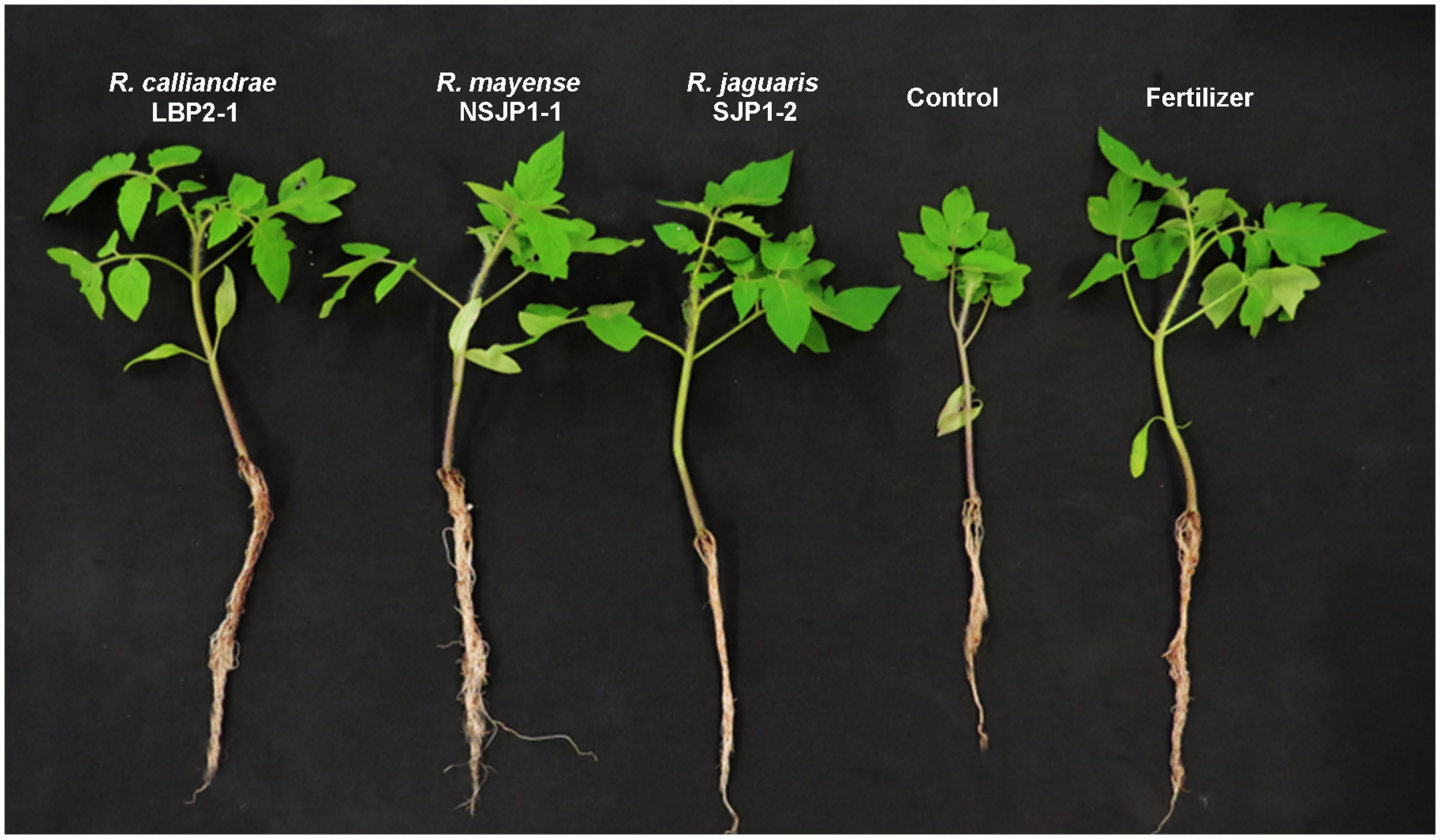
Tomato seedlings after four weeks of being inoculated with the different treatments.

The results of the greenhouse experiments (Table 2; Figure 3) showed that *R. calliandrae* LBP2-1, *R. mayense* NSJP1-1, and *R. jaguaris* SJP1-2 promotes the growth of tomato plants, as inoculation with this strain led to an increase in several parameters related to plant growth. The results of the statistical analysis of these vegetative parameters showed that the plants inoculated with native *Rhizobium* had a significantly higher number of leaves, as well as a larger shoot and root, a higher fresh root weight, besides the increase in the chlorophyll content of the leaves. Figure 3 shows the differences between the seedlings inoculates with the different treatments respect to control. The seedlings inoculated with native *Rhizobium* strains had a higher and more vigorous root mass compared to the negative control plants (without inoculation, without fertilization). Furthermore, plants inoculated with *R. calliandrae* LBP2-1, *R. mayense* NSJP1-1, and *R. jaguaris* SJP1-2 had significantly higher fruits size, as well as the number of fruits per plant. The plants inoculated with the *R. mayense* NSJP1-1 strain had a high potassium content (1.82 mg/g sample) compared to the other treatments. Potassium is an essential nutrient for plant growth and development, and higher levels of potassium can contribute to improved plant health and fruit quality. The treatment NSJP1-1 and SJP1-2 have the highest contents of carotenes (12.31 and 12.28 μg/g) and lycopene (9.87 and 9.89 μg/g sample) in fruits. These results clearly show that inoculation with native *Rhizobium* yields bigger tomato plants of higher quality (Table 3; Supplementary Figure S2).

**Table 2:** Growth parameters and chlorophyll content in tomato seedlings inoculated with native *Rhizobium.*

Treatment	Shoot height (cm)	Root length (cm)	Root FW (g)	Root DW (g)	Chlorophyll (mg/g)
*Rhizobium calliandrae* LBP2-1	20.37^*^ ±1.7 a	20.71 ± 2.5 a	0.23 ± 0.06 bc	0.04 ± 0.019 a	2.48 ± 0.13 b
*Rhizobium mayense* NSJP1-1	21.12 ± 2.0 a	21.45 ± 2.5 a	0.26 ± 0.10 b	0.043 ± 0.018 a	2.92 ± 0.08 a
*Rhizobium jaguaris* SJP1-2	21.18 ± 2.3 a	19.94 ± 2.2 ab	0.26 ± 0.09 b	0.048 ± 0.019 a	2.43 ± 0.17 b
Chemical fertilizer	21.51 ± 2.6 a	20.91 ± 2.8 a	0.38 ± 0.18 a	0.049 ± 0.024 a	2.83 ± 0.07 a
Control	14.92 ± 1.8 b	18.16 ± 2.8 b	0.15 ± 0.06 c	0.037 ± 0.015 a	2.16 ± 0.11 c
HSD^¥^ (*p* < 0.05)	1.879	2.296	0.099	0.017	0.176

*Mean values were obtained from *n* = 25 replicas; ^¥^HSD (Honestly significant difference). Means followed by the same letter are non-significant (*p* < 0.05). FW, fresh weight; DW, dry weight.

**Table 3:** Quality of the harvested tomato fruits.

Treatment	Fruit (g)	Fruit/plant	Potassium (mg/g)	Carotenes (μg/g)	Lycopene (μg/g)
*Rhizobium calliandrae* LBP2-1	26.1 ± 5.1 b	4.0 ± 0.72 a	1.39 ± 0.08 b	12.12 ± 0.056 c	9.34 ± 0.12 c
*Rhizobium mayense* NSJP1-1	28.2 ± 3.9 ab	3.9 ± 0.71 a	1.82 ± 0.03 a	12.31 ± 0.013 a	9.87 ± 0.02 a
*Rhizobium jaguaris* SJP1-2	27.8 ± 5.4 ab	3.7 ± 0.85 a	1.08 ± 0.06 c	12.28 ± 0.021 ab	9.89 ± 0.02 a
Fertilizer	29.7 ± 5.8 a	4.0 ± 0.94 a	1.33 ± 0.05 bc	12.19 ± 0.077 bc	9.52 ± 0.12 b
Control	20.1 ± 4.2 c	3.0 ± 0.79 b	1.08 ± 0.20 c	11.93 ± 0.085 d	9.48 ± 0.11 bc
HSD^¥^ (*p* < 0.05)	2.183	0.713	0.289	0.111	0.177

¥ HSD (Honestly significant difference). Means followed by the same letter are non-significant (*p* < 0.05).

### 3.4. Structure of microbial communities

**Figure 4:**
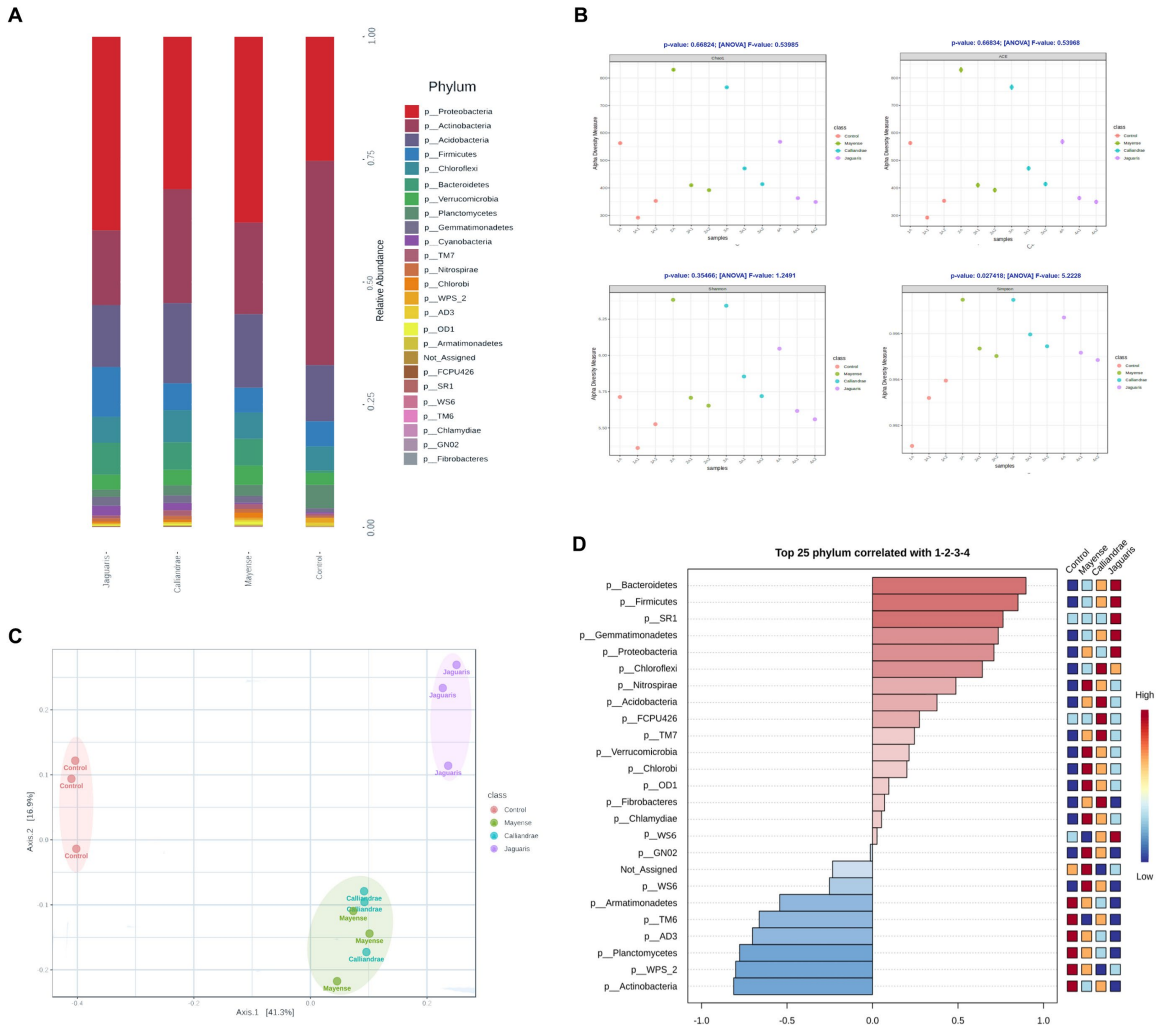
**(A)** Average of phylum composition of obtained sequence on rhizopheric soil biofertilized with each rhizobial strain. **(B)** Analysis of the α-diversity of bacterial communities based on ACE, Chao1, Shannon-Wiener and Simpson indexes (Anova; T-test comparison; *p* < 0.05), and **(C)** β-diversity of bacterial communities based on PCoA showing the distribution of bacterial communities based on their species composition (Distance method Bray Curtis Index, PERMANOVA; *p* < 0.05). **(D)** Enrichment of taxa associated to different treatments.

The dataset contained a total of 7,023 features encompassed by 518,521 filtered reads (1,814,440 before filtered) spread across 12 samples, with a median frequency of 43,210 reads/sample. The results of the study of the rhizobacterial communities showed an increase in relative abundance of Proteobacteria associated with the inoculation of the tested rhizobial strains (Figure 4A). We observed an increase in relative abundance of Proteobacteria and the reduction in Actinobacteria relative abundance, meanwhile, treatment with *R. jaguaris* SJP1-2 also presented an increase of abundance in Firmicutes. Application of *R. calliandrae* LBP2-1 and *R. mayense* NSJP1-1 is associated with an increase of Acidobacteria relative abundance in tomato rhizosphere. Also, we found difference in the Alpha diversity indices in soil samples inoculated with *Rhizobium* strains (Figure 4B). The relative abundance indices Chao1 and ACE increased in treatments with *R. calliandrae* LBP2-1, *R. mayense* NSJP1-1, *R. jaguaris* SJP1-2 but not with statistic differences. A similar behavior occurs with the Shannon diversity index, which increases with treatments with LBP2-1 and NSJP1-1 strains revealed statistic differences respect to control treatment, while not statical differences found with SJP1-2 strain. However, the Simpson diversity index increased in all soil samples inoculated with *Rhizobium* strains compared to the bacterial community control (*p* < 0.05; Figure 4B). The analysis of Beta diversity based on Bray-Curtis Index revealed a similar distribution of bacterial communities in *R. calliandrae* LBP2-1, *R. mayense* NSJP1-1, meanwhile inoculation with *R. jaguaris* SJP1-2 generated changes in bacterial communities distinctive to others (PERMANOVA, *F*-value: 5.4771; value of *p*: 0.001; Figure 4C). These results showed that biofertilization with the selected rhizobial strains induce changes in rhizospheric population in composition and abundance of different taxa. Based on Pearson R correlation index, we determined that inoculation with *R. jaguaris* SJP1-2 was related with the presence of some phyla as Cyanobacteria, Bacteriodetes or Firmicutes and Nitrospirae and Acidobacteria were associated with *R. calliandrae* LBP2-1, *R. mayense* NSJP1-1. In this way, we confirmed that phylum Actinobacteria, Planctomycetes, or WPS_2 between others, had a direct association with control treatment and their abundance is negatively correlated with the inoculation of *Rhizobium* (Figure 4D).

## 4. Discussion

The findings of this study demonstrate that the native strains *R. calliandrae* LBP2-1*, R. jaguaris* SJP1-2, and *R. mayense* NSJP1-1 possess the capacity to solubilize inorganic phosphate, primarily in the form of dicalcium phosphate. Phosphorus is the second most essential nutrient for plant growth (Timofeeva et al., 2022), and these rhizobial strains were isolated from the leguminous plant *Calliandra glandiflora*, which grows in tropical forests in Mexico where phosphorus availability is generally low (Rincón-Rosales et al., 2013). Bacteria mainly utilize the production of organic acids, including gluconic, acetic, glutamic, succinic, malic, or 2-ketogluconic acid, to solubilize mineral phosphorus (Goswami et al., 2016; Billah et al., 2019). These organic acids have chelating properties that aid in the formation of insoluble complexes with metals, leading to the release of phosphate. Several studies have shown that rhizobacteria, including *Rhizobium* spp., are capable of solubilizing phosphorus through the secretion of organic acids (Taktek et al., 2015). Also, these native strains had the capacity to produce siderophores. Siderophores are iron-chelating molecules that play a crucial role in iron acquisition by plants. It is well known that iron is specifically required in N_2_ fixing systems and in the synthesis of nitrogenase enzyme-complex, leghemoglobin, ferredoxin, hydrogenase and cytochrome, and other enzymatic complexes (Datta and Chakrabartty, 2014). Siderophores produced by plant probiotic bacteria can bind to iron ions and transport them to the plant roots, thus promoting plant growth and development. *In vitro*, some *Rhizobium* and *Bradyrhizobium* species produce a variety of siderophores that can suppress some diseases caused by phytopathogenic fungi, which is important in the control of fungal diseases in agricultural crops (Köberl et al., 2016; Ramadan et al., 2016). Auxins are phytohormones that promote plant growth and development. The *Rhizobium* strains LBP2-1, SJP1-2 and NSJP1-1 had the ability to synthesize auxins, such as indole-3-acetic acid (Maurya et al., 2019). Indole-3-acetic acid is a phytohormone that is involved in the regulation and stimulation of plant growth, mainly in the root system. Several species of *Rhizobium* are known for their ability to synthesize this molecule being one of the main functional qualities as plant probiotic (Jiménez-Gómez et al., 2016). Another of the qualities observed in the native bacterial strains LBP2-1, SJP1-2 and NSJP1-1 was the ability to colonize different surfaces competitively. This skill is related with the production of cellulose that is one of the main polysaccharides involved in this mechanisms together other molecules such as exopolysaccharides, lipopolysaccharides and cyclic β-1,2 glucans (Dazzo and Brill, 1979; Robledo et al., 2012) that contribute to the adhesion of the bacteria to root hairs and anchor bacteria to the root surface. The colonization process of *Rhizobium* in non-legume plants, such as tomato, involves a complex chemical interplay between signals from the host plant and chemical responses from the bacteria. Production of polysaccharides and enzymes for cell wall degradation, as well as specific signaling molecules, assist the bacteria in adhering to the plant roots and forming a mutually beneficial symbiotic relationship (Lepetit and Brouquisse, 2023). Also, it was possible to observe the cellulase enzyme activity by native rhizobia. LBP2-1 and NSJP1-1 were the strains that had a greater cellulase enzyme activity when grown in YMA medium supplemented with carboxymethyl cellulose (Supplementary Figure S1). These cellulases are important for the formation of cellulose microfibrils and mediate in the processes of root infection in the bacteria-legume symbiosis. Flores-Félix et al. (2015) reported cellulase activity in *Phyllobacterium* sp. PEPV15, which is essential for cellulose degradation and the formation of microfibrils during the colonization of strawberry roots. The probiotic bacteria produce cellulases that break down cellulose in the plant cell wall, releasing important nutrients such as glucose and other monosaccharides. These nutrients can be utilized by the bacteria for their growth and development, resulting in a beneficial symbiotic relationship for both the plant and the bacteria (Jimtha et al., 2020).

On the one hand, active biofilm formation and root surface colonization are advantageous for plant probiotic bacteria as they enable competition for space, thus isolating other bacteria from their area of action. Simultaneously, the plant can expand its radius of direct interaction through contact with the biofilm (Lugtenberg, 2015). Strains LBP2-1, NSJP1-1 and SJP1-2 exhibited the ability to form biofilms on the roots of tomato plants. Biofilm production is a mechanism that allows bacteria to adhere to the root system of the plant host, thus creating a nutrient exchange surface. Qualitative assays revealed two distinct colonization patterns with variations in intensity and area of colonization (Figure 1A). The differences in biofilm dynamics and production capacity among strains are related to the nature of the exopolysaccharides that make up the biofilm, such as the amount of cellulose and their appearance, which determine the ability to adhere to abiotic surfaces (Kang et al., 2019). Root colonization is a crucial step for promoting plant growth, and this phenomenon can be studied through fluorescence optical microscopy with GFP-labeled bacteria. In this way, we observed that the three rhizobia strains could colonize tomato roots through different colonization strategies. Therefore, these types of studies are very useful in determining the colonization capacities of biofertilizers (Ayuso-Calles et al., 2020; Menéndez et al., 2020; Basu et al., 2021).

The potential as plant probiotic bacteria (PPB) of native *Rhizobium* strains was evaluated in inoculation tests on tomato (*Solanum lycopersicum*) crop. Plants inoculated with the strains LBP2-1, NSJP1-1 and SJP1-2 recorded higher growth and productivity. Also, a positive effect on the size, weight and quality of the fruit was observed. The results of this study indicate that native *Rhizobium* strains have great potential as plant probiotics, with significant benefits for both plant growth and fruit quality. In particular, the inoculation of tomato plants with *R. calliandrae* LBP2-1, *R. mayense* NSJP1-1, and *R. jaguaris* SJP1-2 led to a significant increase in both fruit size and number. Furthermore, the treatments with NSJP1-1 had the highest potassium content, while NSJP1-1 and SJP1-2 had the highest levels of carotenes and lycopene in fruits. These findings suggest that the use of native *Rhizobium* strains as biofertilizers could be a promising strategy to improve tomato production and fruit quality. The efficacy of *Rhizobium* species, such as PPB, has been well-documented in numerous studies that have focused on the biofertilization of vegetable crops (Trabelsi et al., 2011; García-Fraile et al., 2012). For example, inoculation of non-legume plants with *Rhizobium laguerreae* has had a significant impact on the growth, productivity, and phenol content of lettuce (Huang et al., 2007), and it has significantly increased the number of leaves, size, and weight of spinach plants, as well as nitrogen and chlorophyll content (Jiménez-Gómez et al., 2018). Similarly, a significant increase in the growth, number of flowers, and fruits of strawberries (*Fragaria ananassa*) inoculated with the strain *Rhizobium* sp. PEPV16 under microcosm conditions has been reported (Sessitsch et al., 2012). These previous findings support our results obtained from the cultivation of biofortified tomatoes using native *Rhizobium* strains. The LBP2-1, NSJP1-1, and SJP1-2 strains demonstrated their ability as PPBs, which can be attributed to their multifunctional qualities, such as phosphate solubilization, auxin synthesis, siderophore production, among others. Additionally, these strains possess the biochemical capacity to produce cellulose and biofilms, which are key biomolecules for the colonization and infection of the host plant.

Alpha diversity indices were used to determine the abundance and diversity of bacterial communities associated with tomato plants that were inoculated with *Rhizobium* strains. The relative abundance of rhizobacterial communities increased in plants that were inoculated. While *Rhizobium* typically forms a symbiotic relationship with leguminous plants, forming specialized nitrogen-fixing nodules, it can also induce and colonize roots in non-legume plants by producing metabolites such as exopolysaccharides, biofilms, and cellulose, which are key to root colonization. In addition, *Rhizobium* strains can produce other chemical compounds, such as siderophores and antibiotics, that can modify the structure and diversity of the plant microbiome (Robledo et al., 2012). The abundance of species in the tomato rhizospheric community can be influenced by other rhizosphere microbiota, such as *Bacillaceae*, which may compete for root space and nutrients. Therefore, it is important to consider edaphic factors, such as the availability of iron and soil pH, as factors that regulate and limit the diversity of bacterial communities (Han et al., 2020). These findings highlight the potential of native *Rhizobium* strains as biofertilizers to promote plant growth and health, and provide insights into the complex interactions between plant-associated bacteria and their environment. In this study, the bacterial communities associated with tomato plants inoculated with *Rhizobium* strains were found to be dominated by Proteobacteria, Acidobacteria, Bacteroidetes, Verrucomicrobia, and Gemmatimonadetes, which are commonly observed in the root microbiome of different plant species (Sivakumar et al., 2020; Rincón-Molina et al., 2022). *Rhizobium* belongs to the alpha-Proteobacteria group and is known for its multifunctional plant growth-promoting abilities, such as nitrogen fixation, phosphate solubilization, and auxin production. Our observations indicate that native *Rhizobium* strains LBP2-1, NSJP1-1, and SJP1-2 exhibit both plant growth-promoting bacteria (PGPB) and plant probiotic bacteria (PPB) characteristics. These strains can adhere to and colonize the roots of tomato plants by forming biofilms and exopolysaccharides, which are associated with cellulose synthesis. To produce healthy and environmentally friendly food, we need to reduce the use of chemical fertilizers and use plant probiotics in biofertilizers. Our study showed that native *Rhizobium* strains, such as *R. calliandrae*, *R. jaguaris*, and *R. mayense*, have both plant growth-promoting and plant probiotic characteristics. These strains could serve as excellent candidates for biofertilizers, which would improve crop productivity and promote sustainable agriculture practices.

## 5. Conclusion

The results obtained in this work support the potential use for the first time of the native strains *R. calliandrae* LBP2-1, *R. jaguaris* SJP1-2 and *R. mayense* NSJP1-1 as plant probiotic bacteria (PPB) in a tomato crop (*Solanum lycopersicum*). The ability of native *Rhizobium* strains such as PPB to colonize the *S. lycopersicum* rhizosphere and increase plant development, as well as the number and quality of fruits, has been demonstrated. The fruits of the tomato plant inoculated with the native strains of *Rhizobium* present significant increases in the content of potassium, lycopene and carotenes, biochemical parameters that are related to the quality and nutritional value of this vegetable crop. Likewise, it has been determined that inoculation with native strains of *Rhizobium* PPB modify the structure and diversity of the rhizospheric communities, in such a way that the abundance of the phylum Proteobacteria, Acidobacteria, Bacteroidetes, Verrucomicrobia and Gemmatimonadetes increases significantly. Highlighting the increase in the relative abundance of the Proteobacteria, where bacteria of the genus *Rhizobium* is grouped. Therefore, these results demonstrate the potential use of the *Rhizobium* LBP2-1, SJP1-2 and NSJP1-1 strains as a biofertilizer in non-leguminous crops, due to their ability to promote plant development and nutrition in a conventional agricultural system.

## Data availability statement

The datasets presented in this study can be found in online repositories. The names of the repository/repositories and accession number(s) can be found at: https://www.ncbi.nlm.nih.gov/genbank/, PRJNA940440.

## Author contributions

AG-J: laboratory experiments. MR and VR-V: analytical and genetic tools. CR-M and FR-M: investigation. JF-F and LM-G: data analysis. JF-F and RR-R: wrote the manuscript. All authors contributed to the article and approved the submitted version.

## Funding

This research was funded by Tecnológico Nacional de México (TECNM), grant number 16887.23-P and 14094.22-P. JF is recipient of a Marie Skłodowska-Curie grant agreement no. 101003373 from the European Union’s Horizon 2020 research and innovation program.

## Conflict of interest

The authors declare that the research was conducted in the absence of any commercial or financial relationships that could be construed as a potential conflict of interest.

## Publisher’s note

All claims expressed in this article are solely those of the authors and do not necessarily represent those of their affiliated organizations, or those of the publisher, the editors and the reviewers. Any product that may be evaluated in this article, or claim that may be made by its manufacturer, is not guaranteed or endorsed by the publisher.
