# Supplementary material for: Enhance of tomato production and induction of changes on the organic profile mediated by Rhizobium biofortification
Source: Front Microbiol. 2023 Aug 2;14:1235930. doi: 10.3389/fmicb.2023.1235930 (PMC10433389; doi:10.3389/fmicb.2023.1235930)
Supplement: Supplementary file 1 [file Data_Sheet_1.docx]

Supplementary material


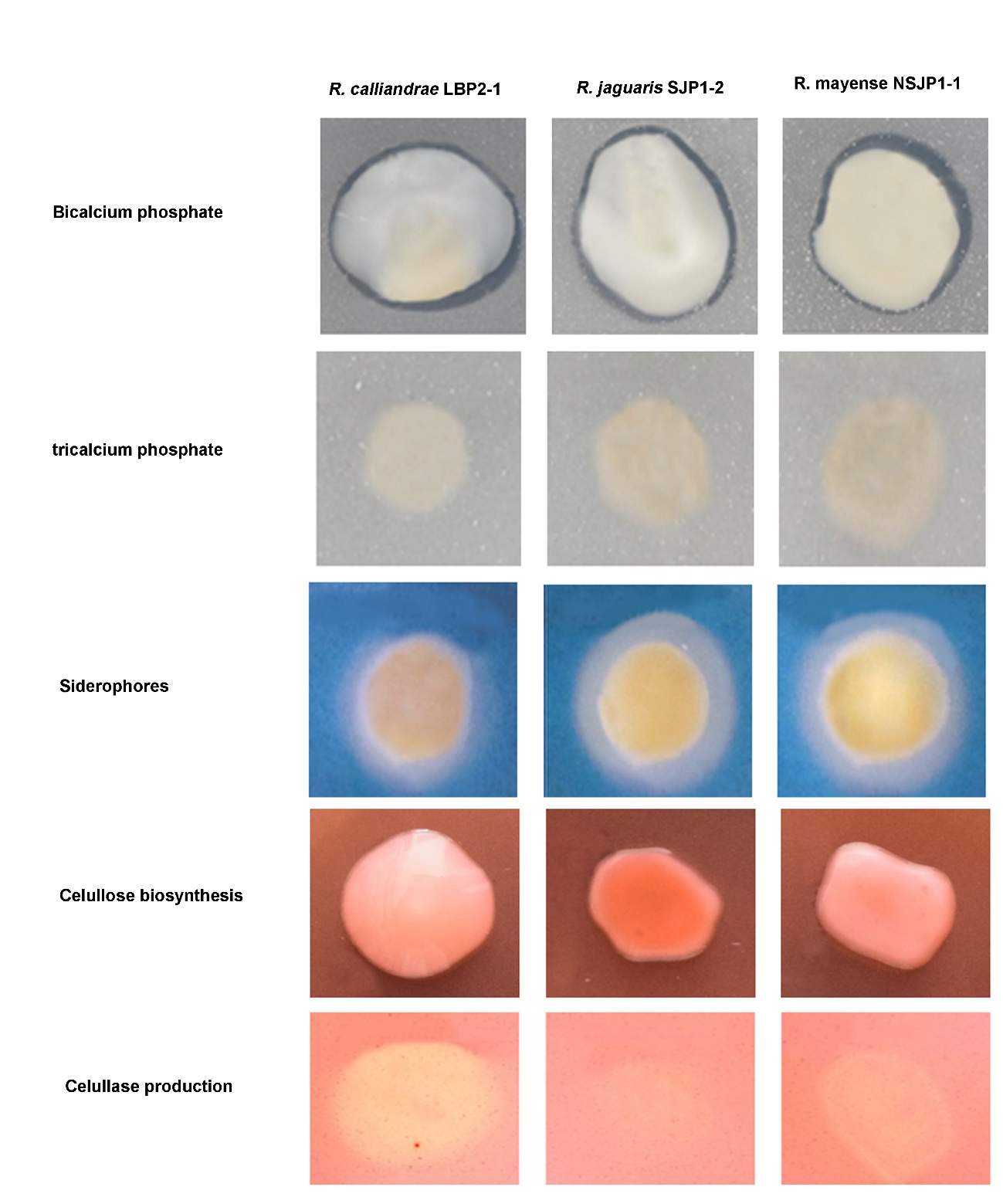


**Figure S1.** Phosphate solubilization halos, siderophores production, cellulose biosynthesis and celullase production by the rhizobial strains.


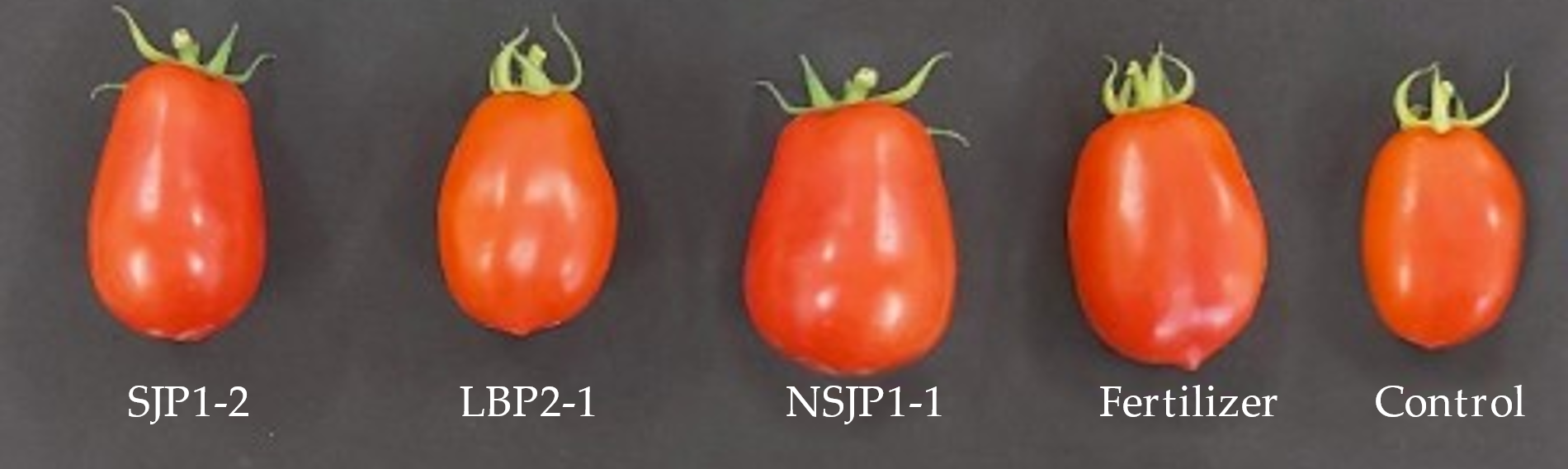


**Figure S2.** Fruits of tomato plants inoculated with native *Rhizobium* from microcosm experiment.
